# A Physically Based Constitutive Model and Continuous Dynamic Recrystallization Behavior Analysis of 2219 Aluminum Alloy during Hot Deformation Process

**DOI:** 10.3390/ma11081443

**Published:** 2018-08-15

**Authors:** Lei Liu, Yunxin Wu, Hai Gong, Shuang Li, A. S. Ahmad

**Affiliations:** 1 Light Alloy Research Institute, Central South University, Changsha 410083, China; Larill@csu.edu.cn; 2 State Key Laboratory of High Performance Complex Manufacturing, Central South University, Changsha 410083, China; csu2019@163.com (H.G.); cmeell@163.com (S.L.); csu_cmee@163.com (A.S.A.)

**Keywords:** 2219 aluminum alloy, constitutive model, microstructural evolution, continuous dynamic recrystallization, hot deformation

## Abstract

The isothermal compression tests of the 2219 Al alloy were conducted at the temperature and the strain rate ranges of 623–773 K and 0.01–10 s^−1^, respectively, and the deformed microstructures were observed. The flow curves of the 2219 Al alloy obtained show that flow stress decreases with the increase in temperature and/or the decrease in strain rate. The physically based constitutive model is applied to describe the flow behavior during hot deformation. In this model, Young’s modulus and lattice diffusion coefficient are temperature-dependent, and the creep exponent is regarded as a variable. The predicted values calculated by the constitutive model are in good agreement with the experimental results. In addition, it is confirmed that the main softening mechanism of the 2219 Al alloy during hot deformation is dynamic recovery and incomplete continuous dynamic recrystallization (CDRX) by the analysis of electron backscattered diffraction (EBSD) micrographs. Moreover, CDRX can readily occur under the condition of high temperatures, low strain rates, and large strains. Meanwhile, the recrystallization grain size will also be larger.

## 1. Introduction

The 2219 Al alloy has long been used in the manufacture of various aerospace components (i.e., oxidizer and fuel tanks) due to its high strength, high fracture toughness, and reliable weldability [1]. During the formative process of the components, the hot working method is of great significance in obtaining the specific shapes and required properties [2]. Moreover, the characterization of flow behaviors in the hot working process largely determines the accuracy of the simulation by finite element method (FEM) [3]. It has been confirmed that the main softening mechanism in hot deformation process involves dynamic recrystallization (DRX) and dynamic recovery (DRV) [4], but the microstructural evolution is often very difficult to illustrate for its complexity [5,6,7]. Therefore, it is extremely important and necessary to investigate both of the flow behaviors and microstructural evolution on 2219 Al alloy during hot deformation [8,9,10].

Over the last decades, many efforts [11,12,13,14,15,16,17,18,19] have been made on the establishment of constitutive models to characterize flow behaviors accurately for various metals and alloys. A mathematical relationship between flow stress and deformation parameters (i.e., temperature, strain rate, strain) is usually used to represent the constitutive model [2]. Firstly, the constitutive model was constructed on the classical Hollomon equation [11] and Ludwik equation [12]. Later, Johnson and Cook [13] proposed the famous Johnson-Cook model, in which work hardening, strain rate, and temperature are taken into account. In addition, Sellars and McTegart [14] proposed another widely applied constitutive model by relating the temperature-compensated strain-rate parameter (i.e., Z parameter) to the flow stress. However, the constitutive model is obviously phenomenological and all the material parameters do not have obvious physical meaning. Recently, according to Mirzadeh et al. [15], a physically based constitutive model was proposed when the glide and climb of dislocations is the main deformation mechanism, in which only two material parameters remain and both of them have physical and metallurgical meaning. To characterize the whole flow curve under different strains, the material parameters are used to be expressed as functions of strain (i.e., strain compensation). This method has been successfully applied for steels [16,17], magnesium alloys [18], commercial-purity aluminum [9], and AA2030 aluminum alloy [19].

During the hot deformation process, DRX often takes place for most metals and alloys. Moreover, different kinds of thermomechanical processing (TMP) may lead to different DRX phenomena, such as discontinuous DRX (DDRX), continuous DRX (CDRX), and geometric DRX (GDRX) [20]. In order to recognize the differences of the three DRX types, many efforts [21,22,23,24,25,26,27,28,29,30,31,32,33,34,35,36,37] have been made on the observation and revelation of microstructural evolution during the hot forming process. DDRX usually occurs with new strain-free grains nucleation and the grains growth in low-stacking-fault energy (SFE) metals and alloys [21]. The investigation of DDRX behavior mainly focuses on the mechanism of nucleation and numerical models, which has been widely applied in different metals and alloys, such as 304 stainless steel [22,23], as-extruded 7075 aluminum alloy [24], and 3Cr2NiMnMo steel [25]. Recently, based on the published researches by Perdrix [26] and Montheillet et al. [27,28], CDRX occurs with high-angle grain boundaries (HAGBs) formed by the progressive lattice rotation of subgrains near grain boundaries during hot deformation. This mechanism has been verified in Mg alloys [29] and Al–Zn alloys [30]. Another transformation mechanism of low-angle grain boundaries (LAGBs) into HAGBs is by the progressive misorientation angles increases. This has also been reported in steel [31] and Al alloys [32]. Based on the mechanisms above, two numerical models of CDRX, the Gourdet-Montheillet model [33] and Toth et al. model [34], are proposed to characterize this process. Apart from DDRX and CDRX, GDRX is a field with less study, but is still observed in some metals and alloys during a large amount of hot deformation, such as commercial-purity Al [35] and Mg–Zn–Zr alloys [36,37]. However, the microstructural evolution with constitutive analysis for 2219 Al alloy under the hot deformation process is rarely reported.

In this study, the flow behavior of the 2219 Al alloy was studied by isothermal compression test, and an effective constitutive model based on physical mechanism was developed. The microstructure evolution during hot deformation was also investigated. Finally, the influence of different TMP conditions on CDRX behavior was discussed.

## 2. Materials and Methods

Isothermal-compression tests were conducted to investigate the flow behaviors and microstructural evolution of aluminum 2219 alloy during hot deformation. The chemical compositions (wt.%) of 2219 Al alloy used in this experiment are shown in Table 1.

**Figure 1:**
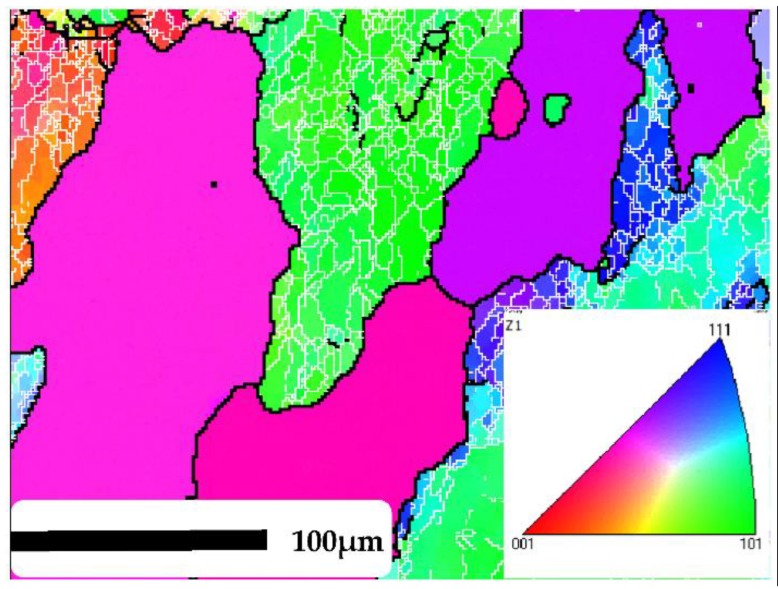
The electron backscattered diffraction (EBSD) micrograph of 2219 Al alloy before deformation.

The samples were cut from wrought billet and machined into a cylindrical shape with a diameter of 10 mm and height of 15 mm by a wire-cutting electrodischarge machine (WEDM, DK7745 by Zhengda (Zhengda Science and Technology Co., Limited, Taizhou, China)). Then, the samples were solution treated at 813 K for 2 h in an electric resistance furnace (SG-XS1200 by Sager (Sager Furnace Co., Limited, Shanghai, China)), followed by water quenching. Hot compression tests were conducted using a Gleeble-3500 thermosimulator (Dynamic Systems Inc., New York, NY, USA.). According to the traditionally used forming temperature for hot working and actual industrial processing, the deforming temperature: *T* = (0.7–0.85) *T_m_* was adopted in this paper. Four different compression temperatures (773 K, 723 K, 673 K, 623 K) and four different strain rates (0.01 s^−1^, 0.1 s^−1^, 1 s^−1^, 10 s^−1^) were applied in the tests; the final true strain is up to 0.9. Each temperature was tested only once under four different strain rates, respectively. Tantalum foil and boron nitride were used to reduce the friction between anvils and the specimen. Each sample was heated to the specified forming temperature at a heating rate of 5 K/s, and then held for 3 min to eliminate the temperature gradient. The true stress-strain data were automatically collected from the computer system during the compression process. After the tests, all the samples were immediately water quenched to preserve the deformed microstructure. Then, the compressed samples were sectioned parallel to the compression axis for subsequent microstructural analysis by the electron backscattered diffraction (EBSD) technique. After the samples were grinded, they were then electropolished in a solution of 30% nitric acid and 70% methanol at 18 V for 50 s. HKL Channel5 software was used to analyze the EBSD data. In all the EBSD images, the LAGBs (orientation angle: 2°–15°) were marked by thin white lines, and the HAGBs (orientation angle: >15°) were marked by the thick black lines. Figure 1 shows the EBSD microstructure of 2219 Al alloy before compression. Coarse equiaxed recrystallized grains and deformed grains with many substructures were both detected.

## 3. Results and Discussion

### 3.1. Flow Behaviors

**Figure 2:**
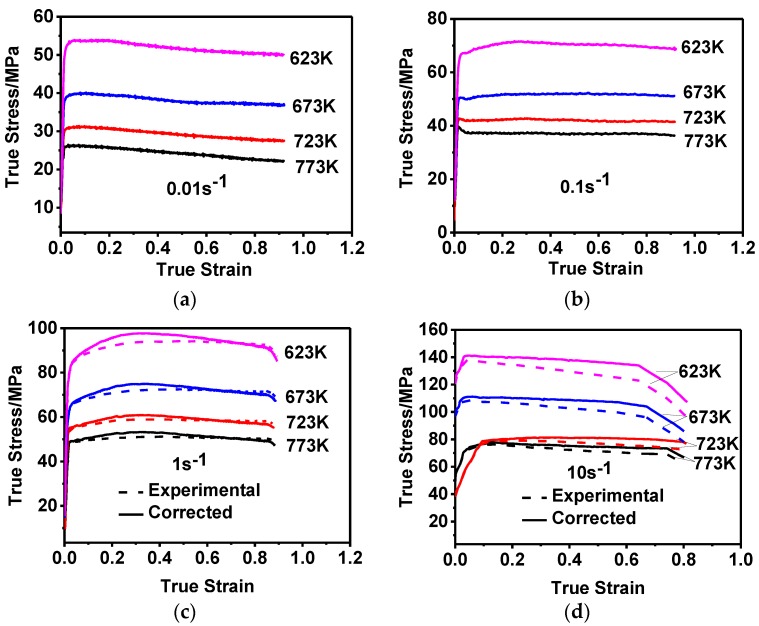
The true strain-stress curves obtained at various strain rates and temperatures of (**a**) 0.01 s^−1^; (**b**) 0.1 s^−1^; (**c**) 1 s^−1^; (**d**) 10 s^−1^.

The true stress-strain curves of the 2219 Al alloy at different temperatures and strain rates are illustrated in Figure 2. Although lubricants were used to reduce friction and isothermal conditions were controlled by the equipment itself, the temperature rise caused by deformation heat and small friction existed cannot be ignored. According to the friction correction criterion proposed by Roebuck [38], the range of B (i.e., barreling coefficient) obtained in this experiment was: 1.02<B<1.09, which means the measured stresses didn’t need to be corrected. In addition, the deformation heat produced as a result of a high strain rate would have a great influence on the flow stress [39], therefore the flow curves under the strain rates of 1 s^−1^ and 10 s^−1^ were modified by temperature compensation according to Humphreys F.J [40]. It can be clearly seen from Figure 2 that the flow stress increased rapidly to a peak value and then slightly fell to a relatively steady value, which shows an obvious characteristic of a single-peak type. Thus, each flow stress curve can be divided into three parts by strains: work-hardening part, softening part, and steady part [41,42]. The mechanism of each part is mainly controlled by the competition between work hardening and DRV and/or DRX during the hot deformation process [43]. For the work-hardening part, the rapid accumulation and proliferation of dislocations leads to obvious work hardening, but DRV develops too slowly. The synthesis result is the rapid increase of flow stress at this stage. For the softening part, high dislocation density largely promotes the development of DRV and the formation of DRX, which has exceeded the effect of work hardening. That is to say, the flow stress will slightly decrease. For the steady part, the multiplication and annihilation of dislocations approximately reached an offsetting state due to the effect of continuous deformation and DRV and/or DRX, respectively, thus, the flow stresses almost remained relatively stable values.

In addition, it is observed from Figure 2 that flow stress exhibited strong dependence on temperature and strain rate. Moreover, flow stress increased with the increase in strain rate and decrease in temperature. This phenomenon indicates that the effect of work hardening is more significant at low temperatures and/or high strain rates. Conversely, DRV and/or DRX developed readily at high temperatures and/or lower strain rates, such as 773 K/0.01 s^−1^, 773 K/0.1 s^−1^, 723 K/0.01 s^−1^. This is because hot compression is a thermal-activation process [5]. Therefore, there will be a larger driving force for the development of DRV and/or DRX due to the easier movement and migration of dislocation and grain boundary [44] at higher temperatures. On the other hand, it will develop more sufficiently to consume more dislocation density at lower strain rate. Thus, either of higher forming temperature and lower strain rate can promote the development of DRV and/or DRX, and finally result in the decrease of the flow stress.

### 3.2. Physically Based Constitutive Modeling

The activation energy means the energy required to overcome the barriers during the deformation process [45]. However, the value of activation energy on 2219 Al alloy (133 KJ/mol) calculated by Arrhenius model exhibits obvious deviation from that of the self-diffusion activation energy on Al alloy (142 KJ/mol) [46]. Some studies [15,46,47,48,49] indicate that the reason for the deviation may be the dependence of Young’s modulus and self-diffusion coefficient on temperature. Therefore, a physically constitutive model was proposed by considering Young’s modulus (*E*) and self-diffusion coefficient (*D*) as functions of temperature. Thus, the mathematical relationship is expressed as follows [47]:

(1) $$\dot{\varepsilon }/D\left(T\right)=B{\left[sinh\left(\alpha \sigma /E\left(T\right)\right)\right]}^{5},$$

where, $\dot{\varepsilon }$ (s^−1^) and σ (MPa) are the strain rate and the flow stress, respectively, *B* and α are the material constants, and the index 5 represents the ideal value of the creep exponent. However, microstructural evolution (i.e., DRX) generally has a certain influence on the value of creep exponent, so it is reasonable to be considered as a variable (i.e., *n*) [39].

Therefore, Equation (1) can be rewritten as:

(2) $$\dot{\varepsilon }/D\left(T\right)=B{\left[sinh\left(\alpha \sigma /E\left(T\right)\right)\right]}^{n}.$$

The relationships between self-diffusion activation energy and temperature, Young’s modulus and temperature can be expressed as [15,47,48,49]:

(3) $$D\left(T\right)=D_{0}exp\left(-Q_{sd}/RT\right),$$

(4) E(T)=2G(1+ν),

where *D*(*T*) is the lattice diffusion coefficient of aluminum alloy, and *D*_0_ is the pre-exponent coefficient of the lattice diffusion (1.7 × 10^−4^ m^2^/s), *Q_sd_* is the activation energy of lattice diffusion (142 KJ/mol), *R* is the universal gas constant (8.31 J·mol^−1^ K^−1^), and *T* is the forming temperature (K). υ is Poisson’s ratio (0.33), and *G* is the shear modulus; its relation with temperature can be expressed as [46]:

(5) $$G/G_{0}=1+\left[\eta \left(T-300\right)/T_{M}\right],$$

where *G*_0_ is the shear modulus at 300 K (2.54 × 10^4^ MPa), *T_M_* is the melting temperature of 2219 Al alloy (916 K), and η indicates the temperature dependence of shear modulus (–0.50). According to the parameters above, *D*(*T*) and *E*(*T*) can be expressed as follows:

(6) $$D\left(T\right)=1.7\times 10^{-4}exp\left(-142000/8.31T\right),$$

(7) $$E\left(T\right)=6.7564\times 10^{4}\left[1-0.5\left(T-300/916\right)\right].$$

In order to obtain the three unknown parameters (α, n, B) in Equation (2), the integrated physically based constitutive equations of the power, exponential and hyperbolic sine laws are summarized and expressed as follows [2]:

(8) $$\dot{\varepsilon }/D\left(T\right)=\{\begin{array}{c} B_{1}{\left[\sigma /E\left(T\right)\right]}^{n_{1}}\\ B_{2}exp\left[\beta \sigma /E\left(T\right)\right]\\ B{\left[\sinh\left(\alpha \sigma /E\left(T\right)\right)\right]}^{n} \end{array},$$

where *B*_1_, *B*_2_, *n*_1_ are material parameters and $\alpha =\beta /n_{1}$.

**Figure 3:**
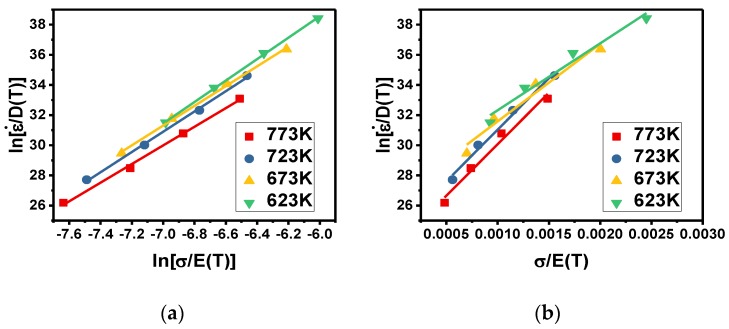
Relationships between material parameters: (**a**) $\ln\left[\dot{\varepsilon }/D\left(T\right)\right]$ against ln[σ/E(T)]; (**b**) $\ln\left[\dot{\varepsilon }/D\left(T\right)\right]$ against σ/E(T).

Based on the power and exponential functions of Equation (8), the material parameters n_1_ and β can be obtained from the gradient of the graph of $\ln\left[\dot{\varepsilon }/D\left(T\right)\right]$ against ln[σ/E(T)] and $\ln\left[\dot{\varepsilon }/D\left(T\right)\right]$ against σ/E(T). These plots are shown in Figure 3 (i.e., taking the strain of 0.5 as an example).

**Figure 4:**
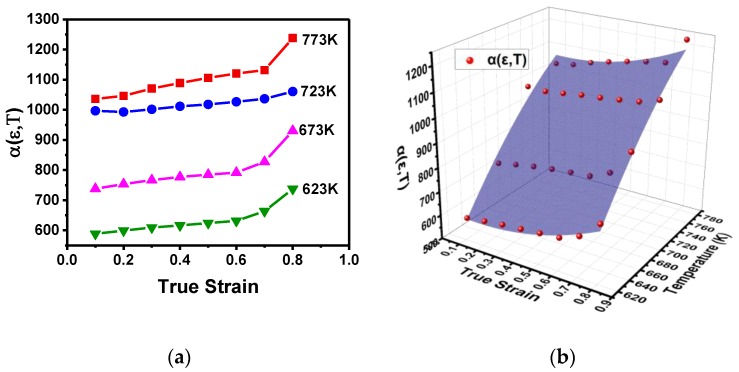
The variation of α: (**a**) at different strains and temperatures; (**b**) 3D illustration and its nonlinear surface fitting.

Therefore, the parameter α can be obtained by evaluating $\alpha =\beta /n_{1}$ at different temperatures and strains; the values are shown in Figure 4a. It can be clearly seen that α is dependent on both temperature and strain and increases with the increase in temperature and/or strain. So α can be expressed as a function of temperature and strain, and its 3D nonlinear surface fitting (Parabola 2D Method) is shown in Figure 4b. Generally, the accuracy of the model’s fitting can be evaluated in terms of correlation coefficient (*R*^2^), which is expressed as follows [39]:

(9) $$R^{2}=\frac{\sum_{i=1}^{N}\left(E_{i}-E\right)\left(P_{i}-P\right)}{\sqrt{\sum_{i=1}^{N}{\left(E_{i}-E\right)}^{2}\sum_{i=1}^{N}{\left(P_{i}-P\right)}^{2}}},$$

where *E_i_* and *P_i_* are the experimental and predicted values of each point, respectively, *E* and *P* are the average experimental and the predicted values, and *N* is the total number of the data sample used. According to the data analysis, α has very good fitting with correlation coefficient *R*^2^ = 0.972; therefore, α can be mathematically expressed as follows:

(10) $$\alpha \left(\varepsilon ,\;T\right)\;=\;-5088.79315-108.92456\varepsilon +13.83184T+319.01959\varepsilon^{2}-0.00757T^{2}.$$

**Figure 5:**
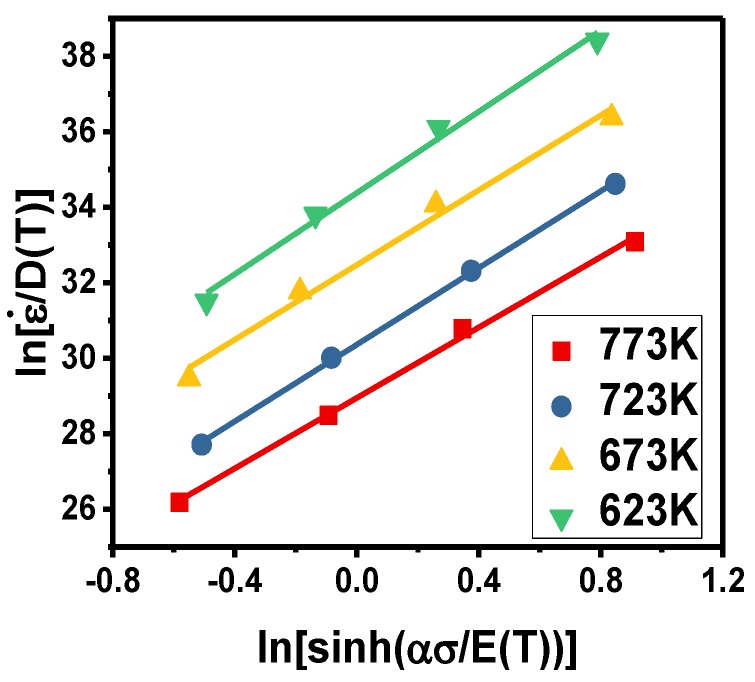
Relationship between $\ln\left[\dot{\varepsilon }/D\left(T\right)\right]$ against ln{sinh[ασ/E(T)]} for different parameters.

According to the hyperbolic sine function of Equation (8), the material parameters *n* and ln*B* can be obtained from the slope and intercept of the graph of $\ln\left[\dot{\varepsilon }/D\left(T\right)\right]$ against ln{sinh[ασ/E(T)]}. The plot under the strain of 0.5 is shown in Figure 5. It can be clearly seen that the fitting lines at different temperatures have an approximate slope (*n*), but the intercepts are completely different. That means n is only a function of strain, while *B* is a function of temperature and strain.

**Figure 6:**
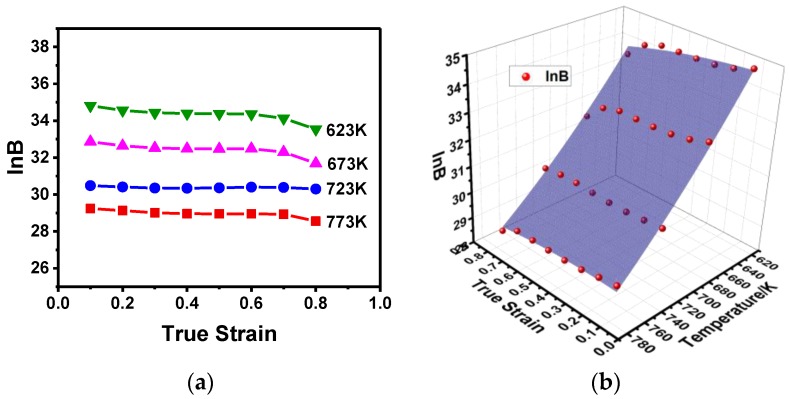
The variation of ln*B*: (**a**) at different strains and temperatures; (**b**) 3D illustration and its nonlinear surface fitting.

The variation of ln*B* under different temperatures and strains is represented in Figure 6a, and the 3D nonlinear surface fitting (Poly 2D) is shown in Figure 6b. According to the data analysis, the correlation coefficient of ln*B* is *R*^2^ = 0.992; therefore, ln*B* is expressed mathematically as follows:

(11) $$\ln B\left(\varepsilon ,T\right)=82.33815-4.1088\varepsilon -0.10644T-0.93899\varepsilon^{2}+4.8375\times 10^{-5}T^{2}+0.00588\varepsilon T.$$

**Figure 7:**
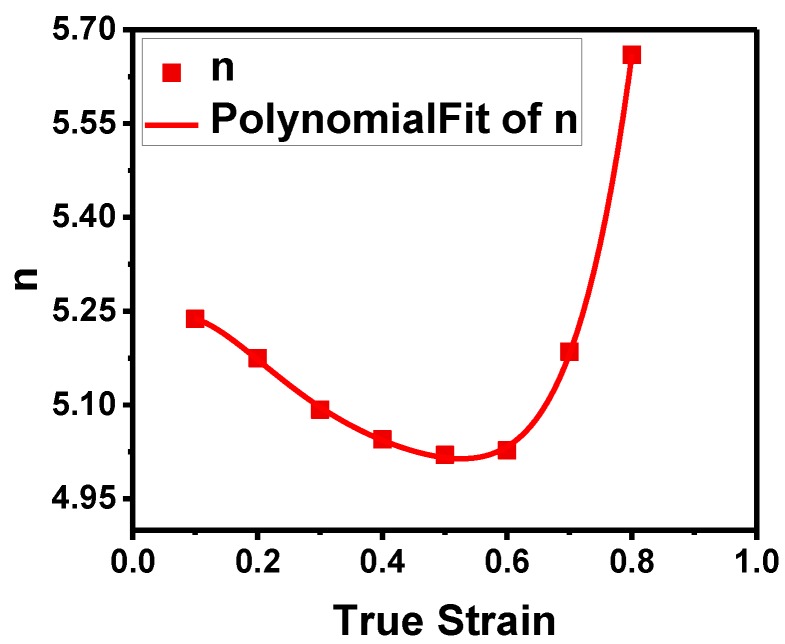
Relationship between true strain and n by polynomial fitting.

The values of n at the different strains are plotted in Figure 7 and fitted by a 5th order polynomial. It can be seen that n is close to 5 and the slight deviation can be attributed to the microstructural evolution. As analyzed from the data, correlation coefficient of n is *R*^2^ = 0.999. Therefore, *n* is expressed mathematically as follows:

(12) $$n\left(\varepsilon \right)\;=\;5.18187+1.83318\varepsilon -17.24749\varepsilon^{2}+52.16674\varepsilon^{3}-72.65807\varepsilon^{4}+39.98397\varepsilon^{5}.$$

The physically based constitutive model of 2219 Al alloy was established based on the governing equations above; from the integrated expression, shown in Equation (13), the flow stress of 2219 Al alloy in the temperature range of 623 K to 773 K, strain rate range of 0.01 s^−1^ to 10^−1^, and the strain range of 0~0.8 can be predicted.

(13) $$\left\{ \begin{array}{l} \sigma =E\left(T\right)/\alpha \left(\varepsilon ,T\right)\mathrm{arcsinh}\left\{exp\left[\ln[\dot{\varepsilon }/D\left(T\right)\right]-lnB\left(\varepsilon ,T\right)]/n\left(\varepsilon \right)\right\}\\ D\left(T\right)=1.7\times 10^{-4}exp\left(-142000/8.31T\right)\\ E\left(T\right)=6.7564\times 10^{4}\left[1-0.5\left(T-300\right)/916\right]\\ lnB\left(\varepsilon ,T\right)=82.33815-4.1088\varepsilon -0.10644T-0.93899\varepsilon^{2}+4.8375\times 10^{-5}T^{2}+0.00588\varepsilon T\\ \alpha \left(\varepsilon ,T\right)=-5088.79315-108.92456\varepsilon +13.83184T+319.01959\varepsilon^{2}-0.00757T^{2}\\ n\left(\varepsilon \right)=5.18187+1.83318\varepsilon -17.24749\varepsilon^{2}+52.16674\varepsilon^{3}-72.65807\varepsilon^{4}+39.98397\varepsilon^{5} \end{array}\right..$$

### 3.3. Verification of the Model

**Figure 8:**
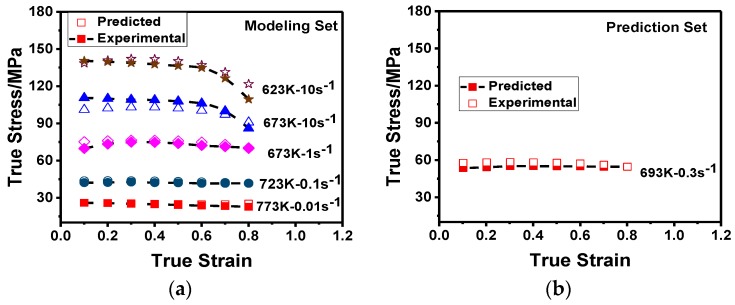

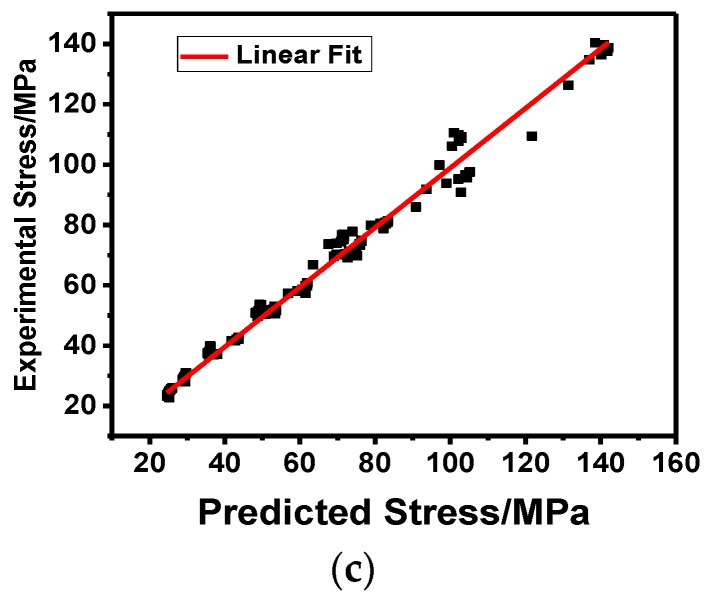
Correlations between the experimental and predicted flow stresses of the physically based constitutive model: (**a**) under modeling set; (**b**) under prediction set; (**c**) by linear fit.

In order to verify the physically based constitutive model, the experimental and predicted stresses under different deformation conditions are compared, as shown in Figure 8. It is obvious that the predicted flow stresses obtained from the physically based model are in good agreement with the experimental flow stresses for both modeling and prediction sets. To quantify the accuracy of the model, the correlation coefficient (*R*^2^) and average absolute relative error (AARE) were determined; the AARE is expressed as follows [39]:

(14) $$\mathrm{AARE}\left(\%\right)=1/N\sum_{i=1}^{N}\left|\left(E_{i}-P_{i}\right)/E_{i}\right|\times 100\%,$$

where *E_i_* and *P_i_* are the experimental and predicted values of each point, and *N* is the total number of the data sample used.

The correlation between the experimental and predicted flow stresses is *R*^2^ = 0.985 and the AARE = 3.88%. Conclusively, the physically based constitutive model can be used to predict the flow stress of 2219 Al alloy during hot deformation with high accuracy.

### 3.4. Microstructural Evolution

#### 3.4.1. The Formation of CDRX

**Figure 9:**
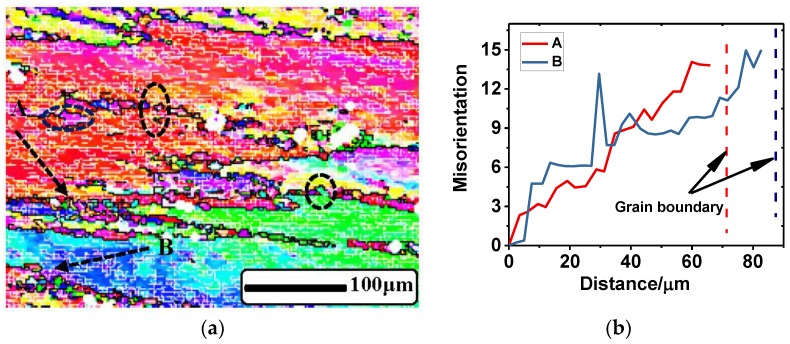
EBSD measurement showing continuous dynamic recrystallization (CDRX) in 2219Al alloy. (**a**) Micrograph of the deformed sample under the condition of 673 K and 10 s^−1^; (**b**) the cumulative misorientation along the dotted line from A and B to the grain boundary.

Microstructure observation was applied to investigate the formation and development of DRV and DRX during hot deformation [4]. Figure 9 shows the EBSD micrograph of the 2219 Al alloy after compression at a temperature of 673 K and strain rate of 10 s^−1^. Comparing with the samples before compression (Figure 1), it is observed that the grains were flattened in the direction of compression and a large amount of substructures with LAGBs were generated in the original grains. The deformed microstructure showed obvious characteristics of DRV. The SFE of the metals and alloys had a significant effect on the softening mechanisms (DRV and/or DRX). For a 2219 Al alloy with high SFE, it is more difficult to dissociate the perfect dislocation into two partials, but perfect dislocation glide, climb, and cross slip can occur easily. During hot deformation, rapid DRV takes place readily, and the stored energy is decreased by rearrangement and annihilation of dislocations, which generally retards the development of DDRX. However, it can be seen from Figure 9a that many fine grains with a size less than 10 μm appeared near the grain boundaries and a few incomplete fine grains (marked with black dotted circles) composed of HAGBs; partial LAGBs were also observed. It is reasonable to deduce that these incomplete fine grains will develop to whole recrystallized grains with continued deformation; that is to say, these partial LAGBs will transform into HAGBs [50]. Therefore, the special formation mechanism of new recrystallized grains is proved to be CDRX rather than the traditional DDRX on 2219 Al alloy during hot deformation [51,52].

Figure 9b exhibits the cumulative misorientation of the two dotted lines (shown in Figure 9a) from the interior of the grain (i.e., A and B) to the grain boundaries, respectively. An obvious increase of misorientation from the interior to the boundary was observed. Thus, as deformation continued, the misorientation of LAGBs near a grain boundary may gradually increase and the LAGBs will transform into HAGBs once the misorientation reached the critical value (15°) [53]. The development mechanism of substructures with LAGBs near the grain boundaries can be ascribed to the progressive lattice rotation [54]. Grain boundary sliding (GBS) was more likely to occur at specifically oriented boundaries, and then these grain boundaries got serrated due to the migration of the HAGBs as shown in Figure 9a. The small serrations on partial boundaries may be eliminated by GBS, but the large serrations or mantles may be gradually formed in the nonsliding boundaries. As GBS can still operate on the mantles, the remaining parts have to sustain the shear effects and distortion begins to occur. As a result, local lattices near the mantles have to rotate and asymmetric bulges are generated. Then, subgrains with intermediate grain boundaries are formed. New recrystallized grains will be formed as long as the partial LAGBs of these subgrains transform into HAGBs [55]. The results of the formation of CDRX are consistent with the study of Yin [50], Shimizu [52], and Beer [53].

#### 3.4.2. The Influence of TMP on CDRX

**Figure 10:**
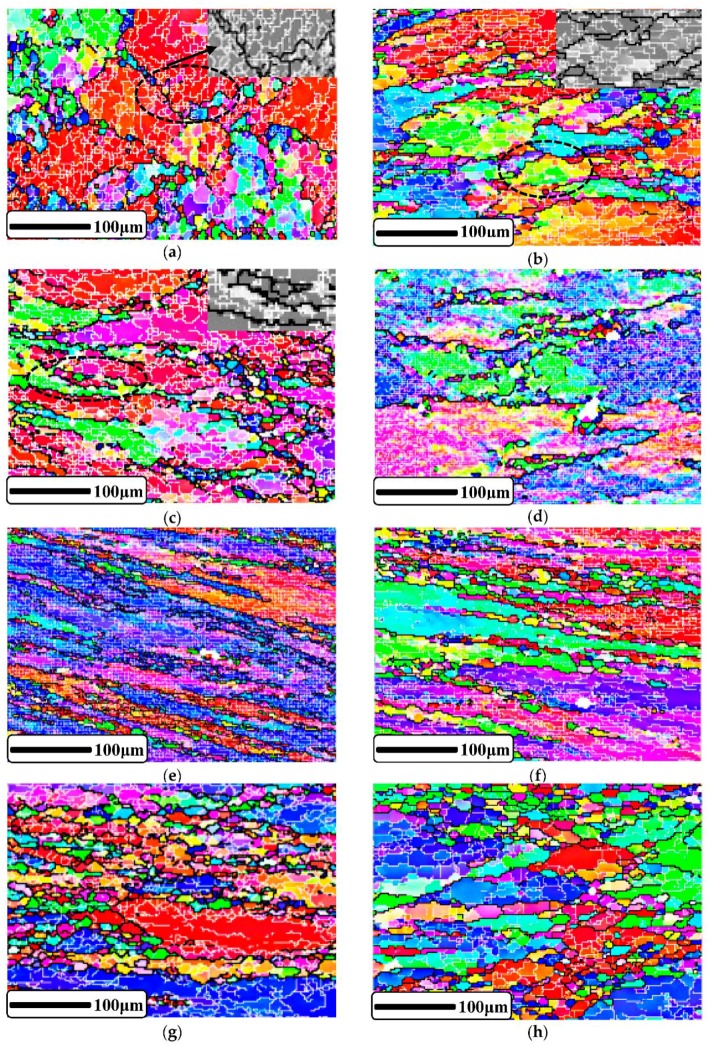
The EBSD micrographs of the deformed samples under conditions of: (**a**) 673 K and 0.1 s^−1^ with the strain of 0.2; (**b**) 673 K and 0.1 s^−1^ with the strain of 0.5; (**c**) 673 K and 0.1 s^−1^ with the strain of 0.9; (**d**) 623 K and 1 s^−1^ with the strain of 0.9; (**e**) 673 K and 1 s^−1^ with the strain of 0.9; (**f**) 773 K and 1 s^−1^ with the strain of 0.9; (**g**) 773 K and 0.1 s^−1^ with the strain of 0.9; (**h**) 773 K and 0.01 s^−1^ with the strain of 0.9.

The EBSD micrographs of the compressed samples under different deformed conditions are illustrated in Figure 10 to investigate the influence of TMP on CDRX. Among them, Figure 10a–c shows the microstructures under the conditions of 673 K, 10 s^−1^, and the strains of 0.2, 0.5, and 0.9, respectively. It can be seen clearly that, as the deformation continued, the original grains were gradually elongated, grain boundaries became more and more jagged, and the amount of small, recrystallized grains near the grain boundaries increased progressively. Thus, the increase in the strain can promote the formation of CDRX. However, the percentage of recrystallized grains is still very small; even at the strain of 0.9, the original grains and their internal substructures occupied the majority, which also proves that the predominant softening mechanism of the 2219 Al alloy during hot deformation was DRV.

In addition, Figure 10d–f shows the microstructures deformed at a strain rate of 1 s^−1^ and strain of 0.9 at different temperatures of 623 K, 673 K, and 773 K, respectively. It can be seen that, as the temperature increased, the amount of recrystallized grains near the grain boundaries gradually increased, and also the grain size gradually increased. This is due to the fact that the mobility of the grain boundaries will increase with the increase in temperature. Moreover, DRX is a thermal-activation process; therefore, there will be a greater driving force to promote the growth of the new grains at high temperature. So, the increase of forming temperature can promote the development of CDRX and growth of new grains. Finally, Figure 10f–h show the microstructures deformed at the temperature of 773 K, strain of 0.9, and different strain rates of 1 s^−1^, 0.1 s^−1^, and 0.01 s^−1^, respectively. It can also be observed that as the strain rate increased, the amount of recrystallized grains decreased, and the growth of new fine grains was greatly restricted. This is because the reduction of forming time restrains the growth of recrystallized grains at high strain rate. Therefore, the increase in strain rates limits the development of CDRX and the growth of its recrystallized grains. The results of the influence of TMP on CDRX obtained are consistent with the study of Wang [51] on AA7050 aluminum alloy.

## 4. Conclusions

In this study, the flow curves under different deformation conditions of 2219 Al alloy were obtained based on thermal-compression tests. The physically based constitutive model was established to describe its flow behavior. The microstructure evolution behavior of the 2219 Al alloy in thermal compression was also studied. The following conclusions can be drawn:

1. The flow stress of the 2219 Al alloy is very sensitive to temperatures and strain rates, and its value decreases with the increase in temperatures and/or the decrease in strain rates.
2. The physically based constitutive model of 2219 Al alloy established is proved to have good predictive performance, which can be used to accurately describe the flow behavior of the 2219 Al alloy in the temperature range of 623 K to 773 K, strain rate range of 0.01 s^−1^ to 10^−1^, and the strain range of 0~0.8.
3. It has been proved that the main microstructure evolution of 2219 Al alloy under hot deformation is DRV and incomplete CDRX. Moreover, CDRX can occur readily at high temperatures, low strain rates and high strains; meanwhile, the recrystallized grains size will also be larger.

## Figures and Tables

**Table 1:** The chemical compositions of 2219 Al alloy used in this test (wt.%).

Si	Fe	Cu	Mn	Mg	Zn	V	Ti	Zr	Al
0.20	0.30	5.8~6.8	0.20~0.40	0.02	0.10	0.05~0.15	0.02~0.10	0.10~0.25	Bal
